# Supplementary figures and images for: Variation in natural infection outcomes and cancer cell release from soft-shell clams (Mya arenaria) with bivalve transmissible neoplasia
Source: PLoS Pathog. 2025 Sep 29;21(9):e1013537. doi: 10.1371/journal.ppat.1013537 (PMC12503300; doi:10.1371/journal.ppat.1013537)

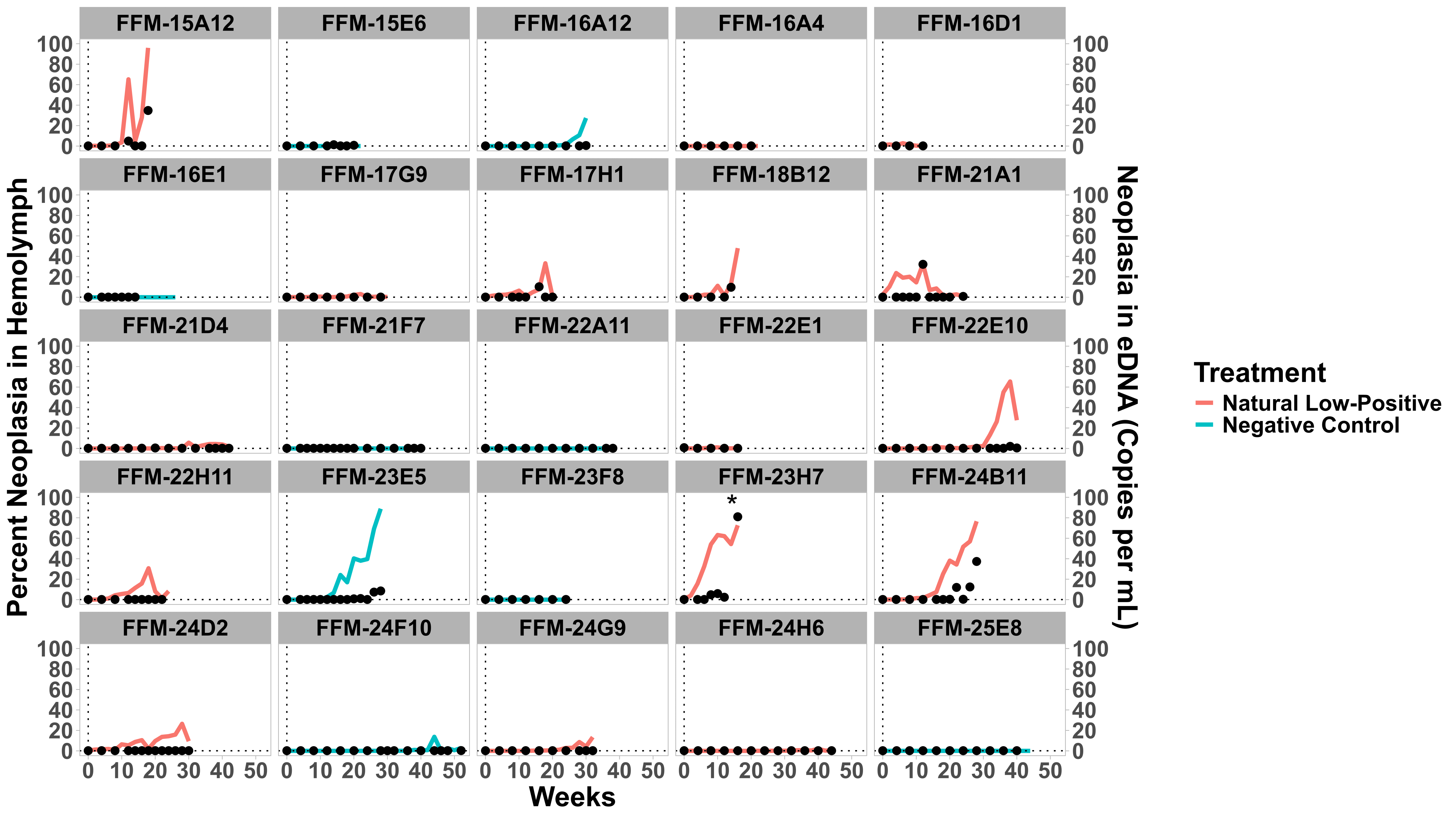

Supplement: S2 Fig — As in the examples shown in Fig 4A–D, eDNA was extracted from tank water collected throughout the study, and MarBTN-specific eDNA was detected using qPCR. Dual axis plots compare the amount of MarBTN cells in the hemolymph (lines, left axis) with quantity of MarBTN-specific eDNA per ml in the tank at the time of collection (black points, right axis). Clams initially diagnosed as naturally low positive (detectible MarBTN < 10% in the hemolymph) have red lines and negative controls clams have blue lines. The animal with clear regression is FFM-21A1. In a second clam (FFM-22H11), the cancer decreased but it did not survive more than 8 weeks after this drop, so under our conservative criteria, we consider it to be progression. All other natural low-positive animals with MarBTN in the hemolymph (n = 8) are animals which progress to death. The slight decrease in MarBTN percent in the hemolymph that is seen in a few cases at the final timepoint before death (FFM-24B11 and FFM-24D2) is likely an artifact due to necrotic release of host DNA into the hemolymph and does not reflect regression of the cancer. Asterisk marks one off-scale eDNA point for FFM-23H7 (1,100 copies/ml at week 14). (TIFF) [file ppat.1013537.s002.tiff]

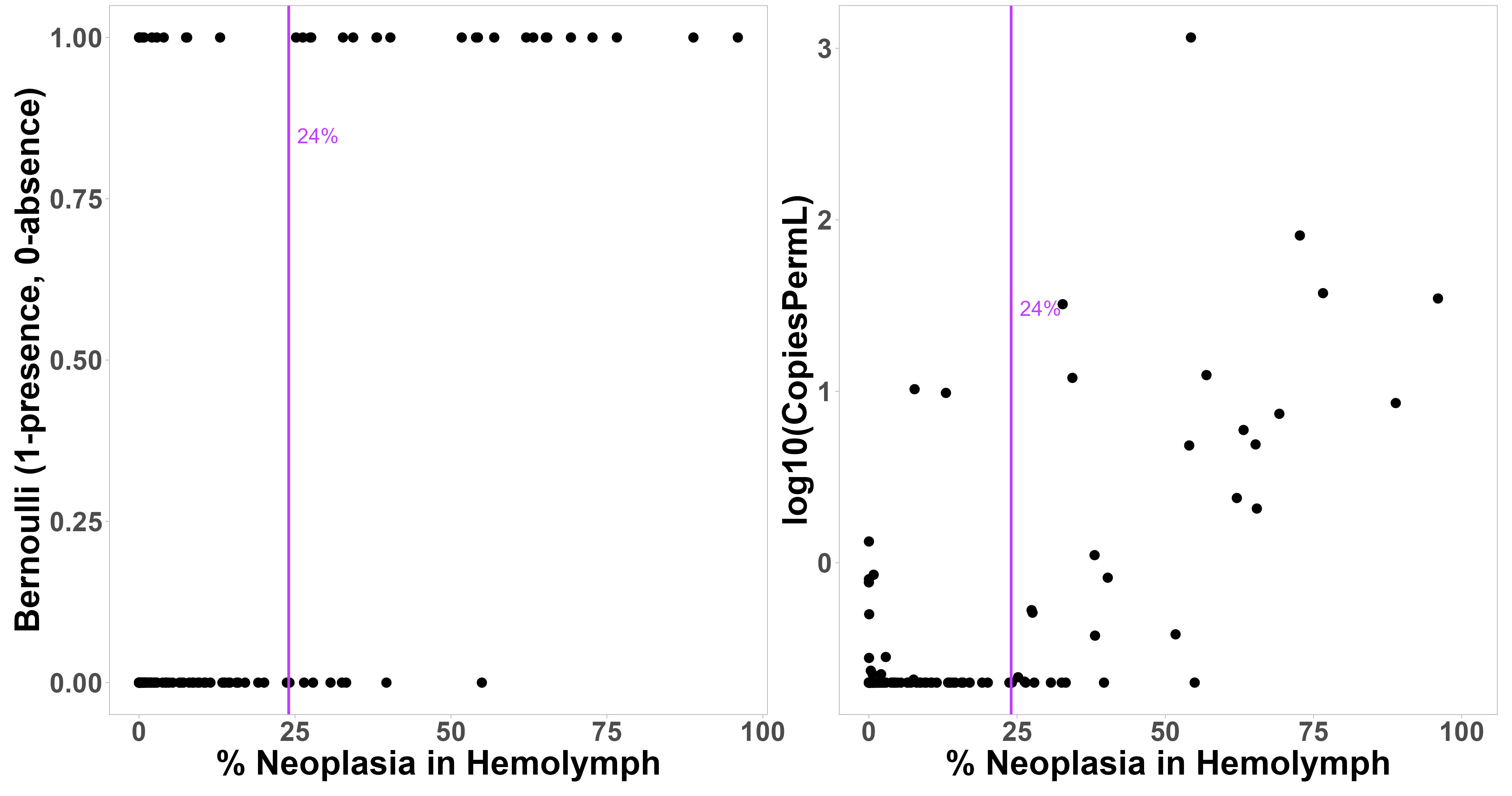

Supplement: S3 Fig — As in Fig 5, for each timepoint for each clam at which both eDNA and hemolymph qPCR results are available, we plot a point comparing the two. The level of MarBTN in the clam is determined by the percent of cells in hemolymph that are MarBTN based on qPCR analysis, and the log transformed copy number of MarBTN-specific eDNA released by clams into their tank water within 24 hrs is plotted on the right panel. The data were also transformed into Bernoulli format (left panel; detectable, 1; undetectable, 0), and 24% was identified as the changepoint (this purple 24% line is plotted on both panels). (TIFF) [file ppat.1013537.s003.tiff]
