# Supplementary material for: Variation in natural infection outcomes and cancer cell release from soft-shell clams (Mya arenaria) with bivalve transmissible neoplasia
Source: PLoS Pathog. 2025 Sep 29;21(9):e1013537. doi: 10.1371/journal.ppat.1013537 (PMC12503300; doi:10.1371/journal.ppat.1013537)
Supplement: S1 Text — (DOCX) [file ppat.1013537.s004.docx]

**S1 Text.** Calculation of equation for estimation of fraction of cancer cells in hemolymph with polyploidy in cancer cells at target site

Terms:

A_C_ = cancer allele number **(qPCR result)**

A_T_ = total allele (control) number **(qPCR result)**

R = Ratio of cancer alleles to total alleles

P_N_ = ploidy of normal cells (usually 2)

P_C_ = ploidy of cancer cells (ie. 4 for MarBTN-USA, 5 for MarBTN-PEI)

Q_C_ = quantity of "cancer alleles" in cancer cells (ie. 2 for both sublineages of MarBTN)

C_N_ = normal cell number

C_C_ = cancer cell number

F_C_ = Fraction of cancer cells **(desired outcome)**

Initial definitions (1-4), then all remaining equations derive from those definitions:

- 1. F_C_ = C_C/_(C_C_ + C_N_) = $\frac{C_{C}}{{(C}_{C} + C_{N})}$
  2. R = A_C_/A_T_ = $\frac{A_{C}}{A_{T}}$
  3. A_C_ = Q_C_C_C_
  4. A_T_ = P_C_C_C_ + P_N_C_N_

- 1. C_C_ = A_C_/Q_C_ = $\frac{A_{C}}{Q_{C}}$
     1. (from #3)
  2. C_N_ = (A_T_ - P_C_C_C_)/P_N_ = $\frac{{(A}_{T} - P_{C}C_{C})}{P_{N}}$
     1. (from #4)
  3. F_C_ = (A_C_/Q_C_)/(A_C_/Q_C_ + (A_T_ - P_C_C_C_)/P_N_) = $\frac{\frac{A_{C}}{Q_{C}}}{\frac{A_{C}}{Q_{C}} + \frac{{(A}_{T} - P_{C}C_{C})}{P_{N}}}$
     1. (substitute 5 and 6 into 1)
  4. F_C_ = (A_C_/Q_C_)/(A_C_/Q_C_ + A_T_/P_N_ - P_C_A_C_/P_N_Q_C_) = $\frac{\frac{A_{C}}{Q_{C}}}{\frac{A_{C}}{Q_{C}} + \frac{A_{T}}{P_{N}} - \frac{P_{C}A_{C}}{P_{N}Q_{C}}}$
     1. (substitute 5 into 7 again)
  5. F_C_ = (A_C_/A_T_)/(A_C_/A_T_ + Q_C_/P_N_ - P_C_A_C_/P_N_A_T_) = $\frac{\frac{A_{C}}{A_{T}}}{\frac{A_{C}}{A_{T}} + \frac{Q_{C}}{P_{N}} - \frac{P_{C}A_{C}}{P_{N}A_{T}}}$
     1. Multiply by (Q_C_/A_T_)/(Q_C_/A_T_)
  6. F_C_ = R/(R + Q_C_/P_N_ - P_C_R/P_N_) = $\frac{R}{R + \frac{Q_{C}}{P_{N}} - \frac{P_{C}R}{P_{N}}}$
     1. (substitute 2 into 9)
  7. F_C_ = P_N_R/(RP_N_ + Q_C_ - P_C_R) = $\frac{P_{N}R}{RP_{N} + Q_{C} - P_{C}R}$
     1. Multiply by P_N_/P_N_
  8. F_C_ = P_N_R/(Q_C_ + (P_N_ - P_C_)R) = $\frac{P_{N}R}{Q_{C}+ \left( P_{N} - P_{C} \right)R}$
     1. (from #11)

For MarBTN-USA: P_N_ = 2; P_C_ = 4; Q_C_ = 2

$$\frac{2R}{2+\left( 2-4 \right)R}= \frac{R}{1-R}$$

For MarBTN-PEI: P_N_ = 2; P_C_ = 5; Q_C_ = 2

$$\frac{2R}{2+\left( 2-5 \right)R}= \frac{R}{1-1.5R}$$
