# Supplementary material for: Variation in natural infection outcomes and cancer cell release from soft-shell clams (Mya arenaria) with bivalve transmissible neoplasia
Source: PLoS Pathog. 2025 Sep 29;21(9):e1013537. doi: 10.1371/journal.ppat.1013537 (PMC12503300; doi:10.1371/journal.ppat.1013537)
Supplement: Attachment — Submitted filename: PLOSPath_reviewresponse_2025-09-10.docx [file ppat.1013537.s010.docx]

PPATHOGENS-D-25-01001
Variation in Natural Infection Outcomes and Cancer Cell Release from Soft-Shell Clams (Mya arenaria) with Bivalve Transmissible Neoplasia
PLOS Pathogens

Dear Drs. Ross, Bhaduri-McIntosh, and Malim,

Thank you for the positive and helpful reviews and for the opportunity to resubmit this manuscript. We believe that we have answered all questions and concerns from the reviewers. All responses to the reviewers’ comments are in indented text below.

Sincerely,

Michael Metzger

**Reviewers' Comments:**

Reviewer's Responses to Questions

**Part I - Summary**
Please use this section to discuss strengths/weaknesses of study, novelty/significance, general execution and scholarship.

Reviewer #1: The manuscript submitted by Giersch, Sevigny et al. is related to the transmissible cancer phenomenon. The study specifically involves bivalve transmissible neoplasia (BTN). BTN is a leukemia-like transmissible cancer, in which the cancerous cells can spread from bivalve to bivalve (clam to clam, in this study). As with other transmissible cancers, the leukemia-like cells are therefore not “self”. Other instances of the phenomenon involve transmission of cells that cause solid tumors in dogs and Tasmanian devils. Thus, BTN is distinct, and can be studied both in the field and in the laboratory.

This report from Giersch et al describes a controlled study of leukemia progression in clams (M. arenaria) naturally infected with MarBTN and provides much needed insights into the variability in uptake and expression. Specifically, the study is significant in that it helps to establish some key parameters of natural infection and disease progression, and does so using naturally infected and uninfected soft-shell clams collected from multiple sites in Maine (which presents its own challenges). From this study, the authors are able to provide insights into variation in progression (and occasional regression), BTN loads in hemolymph, mortality, etc. Among the conclusions, the data suggest that BTN cell numbers have to exceed a certain threshold in the hemolymph before being released into the environment, and the release may occur in “bursts” rather than as a continuous release.

Methodologically, the authors have been rigorous in establishing the experimental and control groups – of some 94 initial clams (47 infected and 47 controls), after strict exclusion the study was initiated with 21 confirmed, naturally low-level infected clams and 39 controls. Three control animals were subsequently found to have low level infection.

The study also includes a brief description of an anecdotal case (lines 219-226).

The introduction also happens to be a strength of this manuscript. Transmissible cancers are unusual as cancers and as pathogens: they are not-quite the same as genetically-induced cancers, but also are distinct from other cellular infectious agents, such as bacteria, parasites and fungi. Thus, this well written, concise introduction is important, as most readers will probably be more familiar with genetically or virally induced neoplasias and/or with more “typical” infectious agents.

Reviewer #2: Re: Review of manuscript # PPATHOGENS-D-25-01001 for PLoS Pathogens “Variation in Natural Infection Outcomes and Cancer Cell Release from Soft-Shell Clams (Mya arenaria) with Bivalve Transmissible Neoplasia” by Rachael M. Giersch*, Jordana K. Sevigny*, Sydney A. Weinandt*, Carissa Mayo, Fiona E. S. Garrett, Karyn Tindbaek, Marisa A. Yonemitsu, Samuel F. M. Hart, Michael J. Metzger.

Summary:
In this manuscript, Giersch and colleagues present a progression-based analysis of a transmissible neoplasia infecting bivalves of the soft-shelled clam species Mya arenaria. Clams were obtained from commercial sources from multiple locations in Maine and status of M. arenaria bivalve transmissible neoplasia (MarBTN) infected/not detectable determined using allele-specific qPCR on DNA extracted from hemolymph. Longitudinal analysis revealed outcomes of infected animals in three main groups including those that progressed to death, long-term non-progressors, and regressors. Analysis of the water for MarBTN by qPCR revealed detectable DNA in levels that correlated with disease progression as inferred via hemolymph. This descriptive study has strengths in supporting the route of transmission via water and variable outcomes including lethality and non-lethality in this species. Major concerns are mostly concerns regarding the overstatement of results. All major and minor concerns of the reviewer are detailed below.

**Part II – Major Issues: Key Experiments Required for Acceptance**
Please use this section to detail the key new experiments or modifications of existing experiments that should be absolutely required to validate study conclusions.

Generally, there should be no more than 3 such required experiments or major modifications for a "Major Revision" recommendation. If more than 3 experiments are necessary to validate the study conclusions, then you are encouraged to recommend "Reject".

Reviewer #1: No major concerns. The study took on the tough task of evaluating disease parameters in naturally collected (and therefore genetically variable and naturally infected) host clams, which means some parameters could not be known precisely. However, the methods are carefully designed to take these challenges into account, and the caveats are acknowledged in discussion.

We thank you very much for your positive review and enthusiasm for the work.

Reviewer #2: Major concerns/comments:
A ‘block’ to MarBTN in the presence of this ‘enzootic’ disease of M. arenaria is referenced several times within the text. There is no evidence of the ability of the clams to ‘block cancer’ (e.g., lines 39, 303, 315, 343-345, 359). Rather, it seems more appropriate that such an ability (or the decrease in MarBTN presence using a sensitive allele-specific qPCR approach) is one possible outcome in the presence of MarBTN and should be explored further. This notion is grossly overstated as currently drafted (particularly in the Abstract and lines 343-345 and 359). Please revise for clarity to the audience.

When we had referenced the “block” to cancer it was meant to convey the observation that cancer progresses in some animals and does not progress in others. We had referred to the non-progression as a block to progression. We have altered the text at all prior mentions of a “block” to clarify that we have observed progression and non-progression and that one possible explanation for this observation is a host-derived block.

Along the same lines, is anything known concerning the presence and spread (geographical areas, season, general proportion infected) of MarBTN? MarBTN is referred to as ‘enzootic’ twice. As currently presented, the significance to the study of this status is unclear but suggests that MarBTN is constantly present but not epidemic. Is this correct? Does the prevalence of MarBTN in the wild reflect the initial detection of animals used in the study? Given the long term relationship (~200 years), are the progression dynamics altered in any detectable way compared to past studies available for comparison? For example, prior to and now post outbreaks mentioned within the Introduction.

The reviewer is correct. MarBTN is constantly present throughout New England, but no notable outbreaks of high prevalence have been observed in these populations for at least 10 years. We have clarified why we use the term enzootic in the introduction. We are using a qPCR method that is able to detect cases with very low levels of cancer cells, so our prevalence numbers are not directly comparable to those done using less sensitive histological methods. We also now elaborate on our comparison of our findings with those findings that were observed when studying DN before it was found to be a transmissible cancer.

Why is 10% used as a cutoff for detection of MarBTN positive clams?

This is an arbitrary value that we chose in order to specifically study disease outcomes starting from early stages. This has been clarified in the text.

Imaging of hemolymph is mentioned in the Methods but images are not provided in the manuscript (perhaps this reviewer has overlooked?). Does the imaging of hemolymph reflect the levels of MarBTN detection over time?

The reviewer is correct. The imaging is consistent with the qPCR results. We now include Supplementary Figure 1, showing examples from a clam from each outcome category. In this new supplementary figure, we present the qPCR results paired with microscopy images taken every 4 weeks (using the clams already highlighted as examples in Figure 4).

What is the typical lifespan (cultured in lab as well as in the wild) of a clam of this species from the 50 mm measurement? Regarding the “limitation of the current study is that we observed a high level of mortality in the uninfected clams in the experimental conditions used here” (lines 323-324), is this consistent with previous work?

It is difficult to determine this exactly, as no studies are available showing the lifespan of soft-shell clams in aquaria, and age and lifespan correlate with latitude, but it is known that they can live 12-20 years. The 50 mm size would be expected to be adults (it is the minimum size for collection in Maine), but still, the mortality we observe in uninfected clams is higher than would be expected in the wild given what we know of their natural lifespans. We now clarify this in the text and include a reference for clam lifespan.

Progressors vs. non-progressors vs. regressors. Other apparent limitations of this study are the lack of knowledge of when the clams were initially exposed to MarBTN and status at the start of the experiment (could a clam already have reached a non-progressor status? Have regressed?). Have the authors tried introducing clams without MarBTN detection followed by quarantine (essentially negative controls) to MarBTN positive tanks? (Wouldn’t such an approach confirm the hypothesis that MarBTN is transmitted via the water column?)..

The experiments proposed would provide additional evidence of MarBTN transmission. We have spent several years working on experimental transmission using a variety of methods, as we agree this is an important study. The results of those experiments are forthcoming, but I can note that co-tanking clams is complicated by the fact that the death of a clam in a small tank can rapidly lead to death of all other clams in that tank. We have had the most reliable success with injection of purified cancer cells, and we are continuing to work on experiments to confirm and characterize water-borne transmission of MarBTN for a future study. Given the long-term follow-up required for any experiments with this pathogen, the study of experimentally transmission will be a separate manuscript. Also, we agree that since we do not observe the exposure, these animals could already have regressed prior to the study, and have included this in the discussion.

Another possible limitation is the collection of the clams, which appears to have been over a timespan ranging from June of 2020 to August 2021 and varies by season. How were the samples selected for inclusion into the study?

There may be differences in seasonality, so we included clams from multiple seasons to prevent this bias. Additionally, for the survival analysis, we made sure to match cases with controls from the same collection so that our survival analysis would not be biased by collection site or date. We now clarify this in the methods.

Why is 50 mm used as a cutoff? The sizes at measurement ranges from 50 to 73 mm. Did the authors query any correlation of MarBTN detection/outcome with size/(age)?

Soft-shell clams are typically regarded as adult at 50 mm (2 inches is the minimum size for collection in Maine). We now note this in the methods. Given the small size range observed here, we did not look for any size correlation.

The ‘anecdotal’ case of the single previously observed regressor maybe requires some clarification. The collection of that clam is prior to those clams collected in the present study (as stated) but appears to coincide with the sample dates from those clams collected in a previous publication, PMID: 35335607. Was the anecdotal clam excluded from a previous study? If so, why?

In the lab there have been multiple experiments to investigate BTN that have been proceeding in parallel. To date, we have not published any study from which this clam was “excluded.” In the study referenced by the reviewer, we were primarily taking samples of MarBTN from a number of heavily diseased clams and testing the effect of different conditions on cell survival in artificial seawater. We were also using several samples to test our ability to detect MarBTN-specific DNA in eDNA extracted from seawater. As observed here, the fraction of MarBTN cells in a disease clam is dynamic, so we would routinely have several diseased animals in tanks in the lab and would conduct the experiments for that paper (and other preliminary studies) using animals that had high numbers of cells at the time they were needed for any particular experiment. This is why we had multiple samples from this clam and noticed that it had regressed instead of progressing to death. Since we did not explicitly follow multiple clams from the same collection, did not follow controls from the same collection, and had not yet fully established our eDNA sampling protocol, we present this animal as an anecdotal “hypothesis generating” case rather than including it in any more carefully conducted study cohort.

Copper et al are said to have also observed the outcomes of “progression to death at high levels of infection, maintenance of the disease at lower infection levels, and remission from lower levels (Cooper et al., 1982)”. Maybe this reviewer is nitpicking, but it seems that previous study would therefore provide a motivation for the current one, rather than having observed the anecdotal regressor clam. After all, there is one additional ‘regressor’ identified in the present study.

While we acknowledge that the Cooper et al paper in itself could have served as a motivation for this study, in writing the manuscript we wished to represent what we have done and what we observed, rather than retroactively changing our motivation to fit a different narrative. We now further elaborate on the Cooper et al study in the discussion to clarify and emphasize its importance.

Figure 1. If including these data, axes and units should be revised to reflect those in later figures presenting progression data, e.g., Figure 2.

We have modified Figure 1 so that the exact axis labels match (i.e. the y axis numbers now include “%” and the lines are of the same thickness). Notably, the range of the x and y axes are now identical across all such figures (0-100% cancer and 0-52 weeks).

Use of the term ‘cancer’ is used somewhat generally throughout the text, and sometimes interchangeably with MarBTN. While it is true that MarBTN is a clonal leukemia-like cancer of the bivalve M. arenaria, the general use for the term ‘cancer’ may at times be misleading to the audience. For example, the study is centered on MarBTN detection via a sensitive qPCR assay. Please revise the text to reflect MarBTN detection and measurements, rather than ‘cancer’ progression dynamics. Is ‘progression’ only indicated by an increase or decrease in MarBTN levels? Are there any other indicators or markers thereof?

We use the term “cancer” to describe MarBTN in the same way that one can use the term “virus” to describe HIV. We have gone through all instances of the word cancer in the text and clarified when necessary, to avoid the implication that we are referring to all cancers instead of referring to this particular cancer. Here we define progression of MarBTN as an increased amount of MarBTN, which then leads to death. We now also clarify that increased cell number is what is meant by “progression,” and we have removed the word in any cases where it was used ambiguously to refer to all outcomes.

Line 36 and throughout the text: “…cancer cell release…”. To this reviewer, the results measure DNA using the allele-specific primers in qPCR, but do not demonstrate the presence of ‘cancer cells’ explicitly. Please revise the wording to reflect what is being shown.

Line 104: the “release of BTN” sounds rather directed as currently stated; suggest “presence of BTN”.

We have clarified in all instances in the text that we are detecting cancer-specific DNA, which is evidence supporting the presence of cancer cells, but we are not directly detecting intact cancer cells themselves.

Line 104: “at different stages of the disease”… By ‘stages’, do the authors just mean the levels of MarBTN as interpreted via qPCR? A statement here reflecting that point near lines 278-279 concerning observations indicating two stages could be of help to the audience and bolster the significance of the results overall.

The word “stages” was unclear in the first instance and has been changed to say “throughout the course of disease.” In the second instance, we are explicitly stating that there is evidence supporting one “stage” in which the animal is exposed but not infectious, and a second “stage” in which they are shedding cells and are infectious. We have attempted to clarify this in the text.

Line 256: The header of this section should be edited to reflect MarBTN levels as interpreted through the qPCR levels rather than ‘Release of cancer cells into water’. In the same section (and as raised by this reviewer elsewhere here), statements should be revised to reflect MarBTN levels rather than high/low amounts of cancer.

We have clarified this header to state “Detection of MarBTN-specific eDNA in tank-water of infected clams correlates with progression of disease” rather than directly implying that we observed intact live cell release. We have also revised the text as suggested above.

Line 350: Suggest removing the word “clear”.

Done.

**Part III – Minor Issues: Editorial and Data Presentation Modifications**
Please use this section for editorial suggestions as well as relatively minor modifications of existing data that would enhance clarity.

Reviewer #1: Line 151-152 “Water samples from the tanks of animals FFM-18E12, FFM-16E1, and FFM-15A12 received a slightly modified protocol and were incubated for 30 min without shaking”. Could the authors include a sentence as to why? Would the reason have any effect on the outcome or interpretation?

After those first samples were extracted, we found (through continued testing), that incubation for 1 hr with shaking gave slightly higher yield than 30 min without shaking (up to a 2-fold increase as detected by qPCR), so the 1 hr with shaking method was used for all further samples. The patterns within each individual would not be altered as all samples from those individuals were analyzed using this method, but the total quantity of MarBTN-specific DNA may be slightly underestimated in these three clams. We now explain this more clearly in the text.

245-249 The authors suggest that the three controls that turned positive reflect a long latent period or period in which infection is localized and not detectable in hemolymph. I wonder if “latent” is the appropriate term here? Does latent include the possibilities of quiescent founder BTN cells and/or ongoing or significant cell division but in a localized environment?
The into and discussion didn’t mention the possibility that variation in the MarBTN population could contribute to differences in progression and outcome. Since the clams were collected from the environment and from different sites, is it possible that another confounding/contributing factor could be variation in the MarBTN cells from clam to clam? This is not a major point, but if this is a possibility or if there is any knowledge related to MarBTN variability, it should be briefly discussed.

We agree with the reviewer, and we now add to the discussion statements clarifying the implications of the term latent, and specific statements in the conclusion regarding the possibility that variation in either the clam population or the MarBTN itself could contribute to the observed variability.

Reviewer #2: Minor Concerns/comments:
Line 40: “This also”… this what?

This has been clarified.
Line 46: “there are three types of transmissible cancers that occur in nature” suggest editing to read “at least three types of transmissible cancers have been observed” or similar.

Done.
Line 58-60: Is there a missing reference for this statement?

We have edited the text to make it clear that this sentence and the sentence before both come from the references that are cited.

Line135: Suggest removing the statement “After data collection, clams were excluded for several reasons”.

We have clarified the statement to read “After data collection, clams were included or excluded from analysis based on the following criteria.”

Line 151: Typo, ‘Water’ should be lower case.

Fixed.

Lines 163-164: The primers are previously published and that citation can be provided.

Done.

Line 168: Suggest editing to remove the last portion of the statement corresponding to “and has not been detected in any healthy clam”.

This is a critical point supporting the use of this primer pair in detection of MarBTN, so the statement has been clarified.

Line 245-247: Suggest removing “healthy” to reflect ‘negative controls’ alone…

Done.

The statement “MarBTN in these three animals shows that there is a significant latent period in natural disease”. Is this true? Do the findings rather suggest that a long latency period may sometimes be present?

I have clarified this statement as suggested to read, “there is sometimes a significant latent period.”

Paragraph near Line 251: The statements should be edited to reflect appropriate tense.

Done.
